# Seismic Behavior of Shear Keys Enhanced with Novel Energy Absorption Devices in Immersion Joints Based on Pseudo-Static Tests

**DOI:** 10.3390/ma15134579

**Published:** 2022-06-29

**Authors:** Xinjun Cheng, Xiang Xu, Liping Jing, Haian Liang, Jie Cui

**Affiliations:** 1 Key Laboratory of Earthquake Engineering and Engineering Vibration, Institute of Engineering Mechanics, China Earthquake Administration, Harbin 150080, China; jlp_iem@163.com; 2 Key Laboratory of Earthquake Disaster Mitigation, Ministry of Emergency Management, Harbin 150080, China; 3 School of Civil and Architectural Engineering, East China University of Technology, Nanchang 330013, China; 2021120402@ecut.edu.cn (X.X.); lianghaiann@foxmail.com (H.L.); 4 School of Civil Engineering, Guangzhou University, Guangzhou 510006, China; jcui2009@hotmail.com

**Keywords:** tunnel engineering, seismic performance of underground structures, immersion joint, earthquake damage, seismic mitigation

## Abstract

Shear keys are usually installed as crucial shear-resistant members of an immersion joint; thus, the mechanical behavior of the shear keys, especially under earthquake loading, deserves more attention. This paper presents a novel arc-shaped energy absorption device developed for shear keys. In order to verify the seismic performance of shear keys strengthened by the arc-shaped energy absorption devices, a series of pseudo-static tests were conducted, in which different axial pressures (300 kN, 400 kN) were also taken into consideration. The testing results indicated that failure mode of the shear key enhanced by the energy absorption devices was a synthesis of the oblique shear failure of the rubber blankets, the buckling of the energy absorption devices, and the concrete fracture of the shear key. In view of load-displacement hysteretic curves of testing specimens, loops of the reinforced shear keys were plumper than those from a traditional shear key. In addition, the load-bearing capacity (cracking load, yield load, peak load, and failing load) differences of the shear keys with and without energy absorption devices reinforcement under the same axial pressure were 33.0%, 36.7%, 26.0%, and 23.6%, respectively. The maximum equivalent viscous coefficient values of the shear keys with and without energy absorption devices reinforcement were 0.37, 0.38, and 0.32, respectively. The arc-shaped energy absorption devices can contribute to the hysteretic behavior of the shear keys. However, the axial pressure had a positive influence on the load bearing capacity, accumulated energy absorption capacity, and initial stiffness of the shear keys. In contrast to that, the axial pressure had negative influence on ductility ratio of the reinforced shear keys (equivalent viscous coefficient values of two enhanced shear keys were roughly equal). A reasonable stiffness scheme of an energy absorption device should be given attention during the anti-seismic design of an immersion joint. The study can provide scientific support for further study on the seismic responses of immersion joints and promote the application of earthquake control technology in immersed tunnels.

## 1. Introduction

With worldwide environmental change and the further agglomeration of population and resources in urban agglomerations, the resilience of cities is becoming increasingly important. Currently, there is no comprehensive disaster prevention plan for urban agglomerations, natural disaster prevention, and risk control. The coping strategy research is still weak. As an effective part of the urban underground transportation system, immersed tunnels play an increasingly significant role. Buried on seabed or riverbed, immersed tunnels are classified as shallow-buried rectangular structures, which may suffer serious damage during earthquake events. As the stiffness of the immersion joint is usually smaller than that of the immersed tunnel element, imposed deformations would be focused on the immersion joint. Therefore, it is urgent to solve seismic damage problems and seek effective ways to ensure the security and sustainable development of immersed tunnels.

The development of immersed tunnels can date back to 1896, Gursoy minutely described the developments and reformations of immersed tunnels in America [1]. Hu [2] introduced relevant research on the Hong Kong–Zhuhai–Macao Bridge (HZMB) tunnel project and then put forward engineering schemes for the project. Su et al. [3] presented the Hong Kong Zhuhai Macao tunnel project from the client’s perspective. Based on the Hong Kong Zhuhai Macao tunnel project, Song et al. [4] conducted a numerical analysis to study the rationality of a semi-rigid tunnel element scheme. It was found that the semi-rigid tunnel element has superiorities in regard to reinforcing the sealing and shear resistance capacity of immersion joints. Bergsma et al. [5] described that ice formation must have far-reached impacts on the construction of an immersed tunnel. They also proposed that everyone should notice that temperature-related aspects, i.e., rubber gasket damage, thermal strains, and other geotechnical problems may also come up. Zhou et al. [6] developed a novel type of ballast water system with flexible water-filled containers to overcome the shortcomings of the traditional ballast water system. The system has been adopted in the Yuliangzhou immersed tunnel construction.

The evaluation of the stability and steady state of an immersed tunnel is extremely significant. According to the fuzzy analytic hierarchy process method, Wu et al. [7] carried out a hydrodynamic scale-model test to investigate the effects of motion response on the safety of an immersed tunnel element during the immersion process. Guo et al. [8] studied thermal stress and fire damage of an immersed tunnel structure and pointed out that having or lacking thermal insulation was crucial to tunnel structure. Xu et al. [9] established the hierarchical structure of the health index and then put forward a modified evaluation model. The proposed model about the health state of immersed tunnels was verified through field data of the Nanchang Honggu immersed tunnel. Based on the Biot dynamic consolidation theory, Zhao et al. [10] proposed a cyclic characterization framework for the elastoplastic consolidating feature of the liquefiable seabed around immersed tunnels under combined loads. Furthermore, to comprehensively analyze the seismic performance of immersed tunnels in the liquefiable seabed, a simplified soil hysteresis model for saturated sand [11] and a fully coupled flow deformation model [12] were also recommended. On the basis of the Anatasopoulos method [13], Zhang [14] established a spatial nonlinear model with a consideration of temperature and prestress to synthetically study the seismic performance of an immersed tunnel. It was found that temperature falling load has adverse influences on the seismic behavior of immersed tunnel. Assuming that the push-pull deformation would dominate the axial strain of an immersed tunnel, an estimation of the influence of flexible immersion joints on the seismic performance of immersed tunnel was investigated by Hamada [15]. Yuan et al. [16] proposed that the dynamic responses of tunnel elements and immersion joints can be calculated by using the matrix method based on the multibody dynamics [17]. Based on numerical simulation, Ding et al. [18] investigated the seismic performances of an immersed tunnel project and pointed out that a three-dimensional contact model can effectively improve computational efficiency. Yu et al. [19] put forward a closed-form solution of an immersion joint on the basis of a benchmark model and a refined immersion joint model.

In retrospect, there have been a certain number of laboratory experiments on the mechanical characteristics and seismic responses of immersed tunnels. Yuan [20] conducted a series of scale model experiments on immersion joints, and compression-shear forces induced by earthquakes were considered. They pointed out that immersion joint specimens underwent a nonlinear stage and a quasi-linear stage, and damage occurred at the vertical shear keys on the sidewall. Xiao et al. [21] investigated the compression-shear performance of a scaled immersed joint model by using a static test. Yu et al. [22] conducted an experiment on the mechanical characteristics of an immersion joint model and proposed that the ranges of flexural and shear stiffness ratio were 1/1161 to 1/25 and 1/76 to 1/24, respectively. Wang et al. [23] carried out a series of shaking table tests to study the seismic response of soil-immersed tunnel systems with a consideration of the effect of the overlying water and presented that water has a great influence on the transverse responses of the soil-immersed tunnel system. Based on a series of non-uniform shaking table tests for immersed tunnels, Yu et al. [24] proposed that the seismic responses of immersed tunnels under non-uniform vibrations were greater than those under uniform vibrations. Chen et al. [25] performed non-uniform shaking table tests of an immersed tunnel model with a consideration of a soil site and deemed that the model design method proposed in their study could satisfy the similarity relation of the immersed tunnel for shaking table experiments.

Li et al. [26] developed a novel type of shear key using hybrid fiber-reinforced concrete (HFRC). To investigate the seismic performances of the HFRC shear keys, a series of cyclic loading tests were conducted. The influences of mixture proportions of the concrete on the mechanical performances of testing shear keys were analyzed. The testing results indicated that the HFRC shear keys exhibited better mechanical performances than ordinary shear keys. An analytical model used to calculate the ultimate load-carrying capacity of the HFRC shear keys was finally proposed. In addition, the energy dissipation method is an efficient way to avoid massive damage to immersed tunnels. On the basis of metal buckling dampers, Yu et al. [27] investigated the vibration reduction performance of an immersion joint accompanied by dampers and found that bending moments and hysteretic areas of the testing joint were increased dramatically.

**Figure 1:**
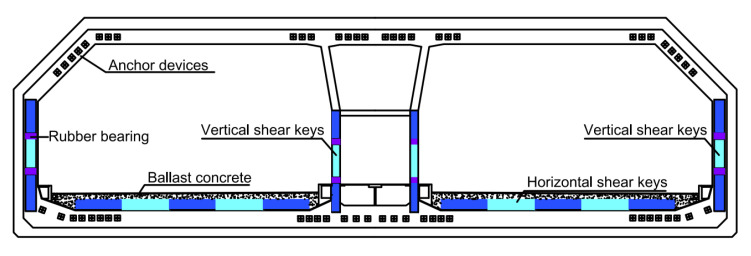
Shear keys in the immersion joint [2,28].

Shear keys are the main bearing carriers of immersion joints. When subjected to seismic loading, shear keys can resist shear forces in the transverse section. The studies mentioned above were mainly focused on the overall performances of immersed tunnels under seismic vibrations or static loads and the mechanical characteristics of immersion joints under different static loads. The information about the seismic behaviors and anti-seismic investigation on important components of immersion joints, i.e., vertical and horizontal shear keys (as shown in Figure 1) is still sparse. Therefore, analytical and experimental investigations on the seismic responses of the shear keys in immersion joints are worth conducting.

To illustrate the effectiveness of the arc-shaped energy absorption device and investigate the axial pressure on the seismic behavior of the shear key enhanced by the arc-shaped energy absorption devices, a series of pseudo-static tests were conducted. Firstly, a novel arc-shaped energy absorption device was developed for shear keys. Then, a test program that consisted of 2 shear key models reinforced by the arc-shaped energy absorption devices was also prepared, and all the shear key models were 1/4 of their sizes in engineering. Different axial pressures (300 kN, 400 kN) were adopted to simulate different water pressures on immersion joints at diverse water depths. Cyclic horizontal displacement loading was mainly considered. Based on experimental results, the failure mode, ultimate carrying capacity, hysteretic characteristics, and stiffness degradation were synthetically analyzed, and the influence of the axial pressures on the seismic performance of the shear key models was also discussed.

## 2. Development of a Novel Arc-Shaped Energy Absorption Device

According to [22], the minimum transverse shear stiffness ratio of immersion joint to immersed tunnel body itself is only 1/76, which indicates that immersion joints are the weak portions of an immersed tunnel. When subjected to severe earthquakes, prominent damages i.e., opening, yield stress, concrete cracks, and permanent deformation of the water sealing profile may occur at the joint, which can cause immeasurable losses to the society. Therefore, some seismic measures should be taken to dissipate the input seismic energy at the immersion joint. The principle of the structural energy dissipation method is to dissipate part or all of the dynamic energy of the structure by taking advantage of the plastic deformation of a damping device in the earthquake process. In that way, the seismic forces of the immersed tunnel structure can be effectively reduced, and the seismic safety of the immersed tunnel structure then can be ensured [29]. Based on the method mentioned above, the main authors of this research developed an arc-shaped energy absorption device by using Q235 mild steel, which was applied to the shear keys in immersion joints.

**Figure 2:**
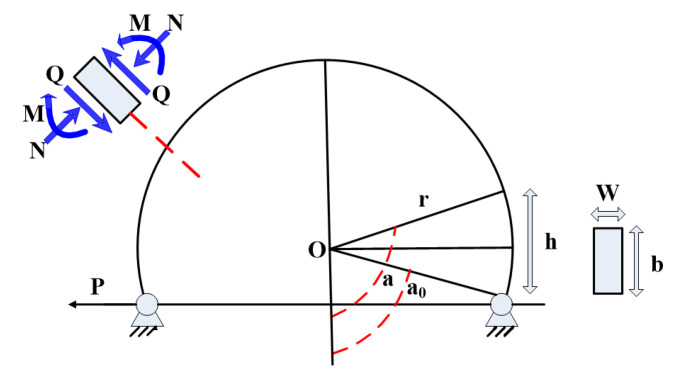
Mechanism diagram of the energy absorption device.

**Figure 3:**
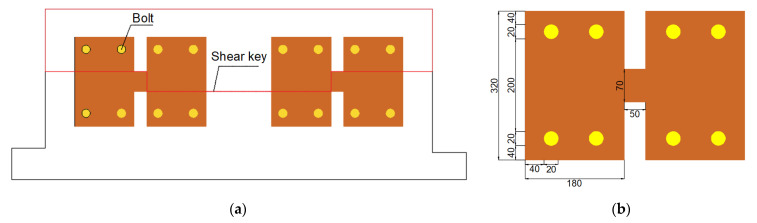
Schematic diagram of the energy absorption device: (**a**) Connection schematic diagram; (**b**) Dimension parameters of the energy absorption device.

As shown in Figure 2, the arc steel metal plate is required to bear the horizontal reciprocating load induced by seismic excitations. The arc-shaped energy absorption devices can be installed at both ends of shear keys using high-strength bolts (as shown in Figure 3). Considering the fact that the outer walls of the immersed tunnel engineering project are located in an aquatic environment, it is difficult to replace the energy absorption device outside the tunnel, so the installation site of the arc-shaped energy absorption device should be inside the tunnel. The mechanical parameters of the energy absorption device are shown in Table 1.

## 3. Pseudo-Static Tests on Seismic Performance of Shear Keys Reinforced by the Arc-Shaped Energy Absorption Devices

### 3.1. Details of the Testing Model

In order to verify the effectiveness of the arc-shaped energy absorption device and investigate the influence of the axial pressure on the seismic behaviors of shear keys, comparative tests must be carried out. Given that previous research launched by our team has presented the seismic performances of a traditional shear key model (hereafter referred to as S0) subjected to horizontal cyclic loading [30], two specimens enhanced by the arc-shaped energy absorption devices were fabricated. Table 2 shows the specific parameters of S1 and S2. It should be noted that the materials of S1 and S2 must be kept the same as S0. As S0 has considered the axial pressure of 300 kN, so the same axial pressure should be applied to S1.

**Figure 4:**
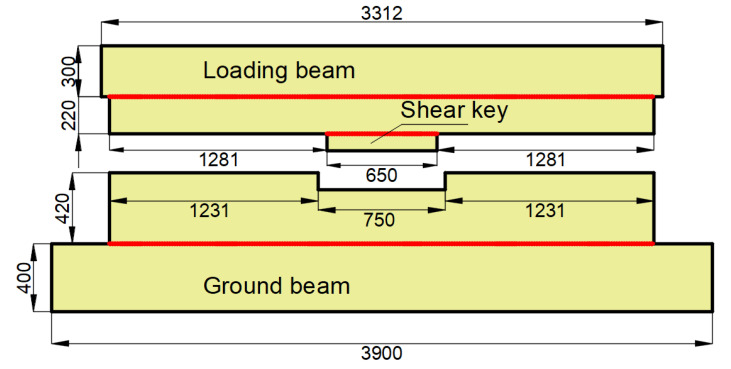
Elevation diagram of immersion joint model.

**Figure 5:**
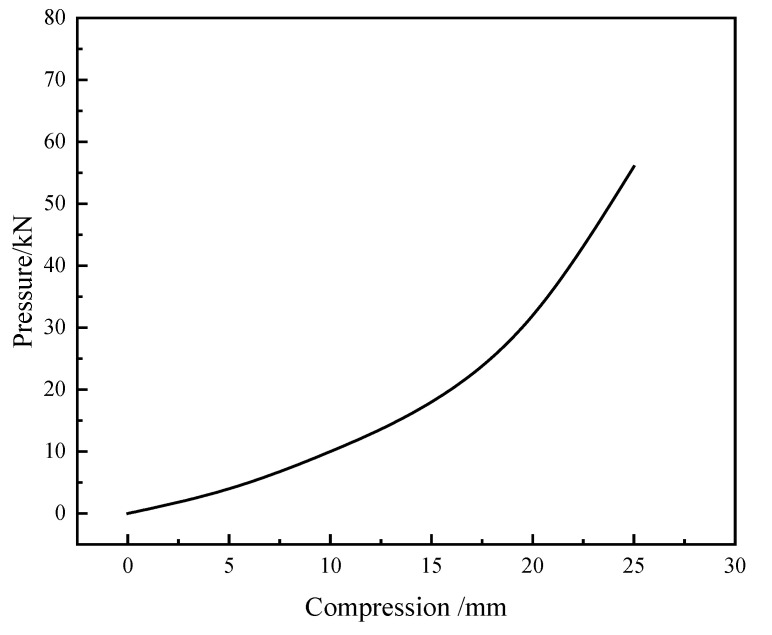
Mechanics performance curve of rubber material used in the study.

Each specimen consisted of an upper “T”-shaped portion with a shear key and a bottom “U”-shaped portion. As shown in Figure 4, the upper portion was made of a loading beam with lengths of 3312 mm, widths of 387 mm, and heights of 300 mm, and a shear key with lengths of 650 mm, widths of 187 mm, and heights of 100 mm, and the bottom segment was made of a ground beam with lengths of 3900 mm, widths of 787 mm, and heights of 400 mm, and a “U”-shaped groove with lengths of 750 mm, widths of 187 mm, and heights of 100 mm. Eight anchor holes with diameters of 100 mm were arranged at both ends of the ground beam to fix the specimen. The gaps between the shear key and groove were filled by high damping rubber blankets. As shown in Figure 3, four high-strength bolts should be set at both the left and right sides of each gap to connect the energy absorption device. Both specimens were prepared with the same materials, which were consistent with practical engineering. Concrete with a strength grade of C50 was adopted in the study, and the grade of the main steel bars used in the models was HRB400. The shear stiffness of the rubber material is 1.06 MPa, and the performance curve of the rubber material used for the rubber blankets in the research is shown in Figure 5. According to the compression and load-bearing capacity abilities of the rubber material, the rubber blankets used in the study can satisfy the flexible feature of an immersion joint.

### 3.2. Test Setup and Sensors Arrangement

**Figure 6:**
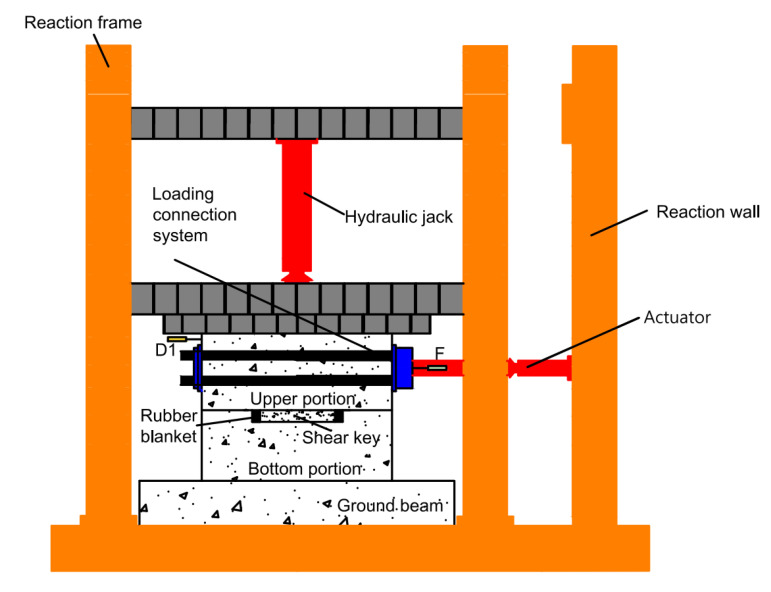
Setup for the tests.

**Figure 7:**
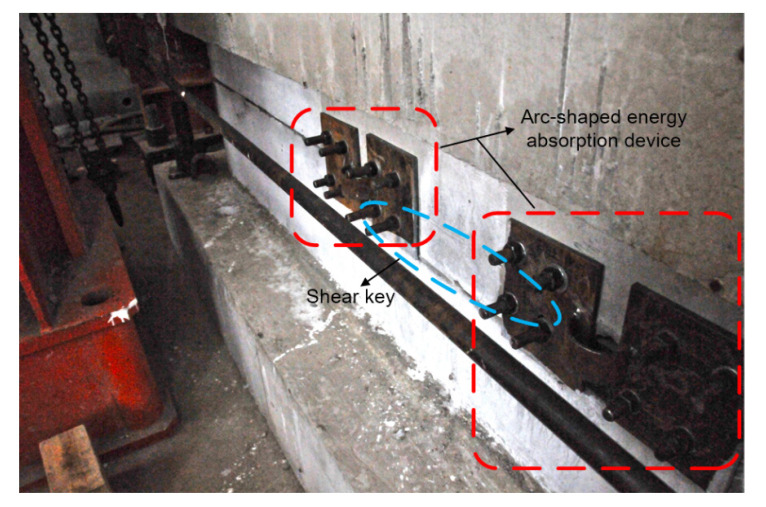
Assembled specimen.

Figure 6 shows test setup and sensors arrangement for pseudo-static tests for the shear keys. For testing models, the bottom portion was first fixed to the ground by high-strength screws. Then the upper portion was installed on the bottom portion. The horizontal loading connection system was comprised of a steel plate connector at one end of the loading beam of the upper portion and a perforated plate at the other end of the upper portion, four high-strength screws, and a horizontal actuator which was fixed on the reaction wall. Two steel plates were connected by four high-strength screws passed through the screw holes. The horizontal actuator and loading beam can then be effectively connected through the “bolted-steel-structure” method. Furthermore, there also existed a vertical hydraulic jack installed on the reaction frame system, and the axial pressures were applied to the specimen through this jack. To prevent model slippage, two jacks were installed at both ends of the floor beam to reinforce the ground beam. Figure 7 shows a typical assembled specimen.

The load-displacement curves of the specimens were measured during the experiments. Figure 6 presents the transducers arranged on the specimens. Linear varying displacements transducers “D1” (namely LVDTs) were fixed at the “T”-shaped portion to obtain horizontal displacements. The horizontal reaction forces of the specimens at diverse displacement loading stages were recorded by the load transducer “F” installed on the horizontal actuator.

### 3.3. Loading Scheme

**Figure 8:**
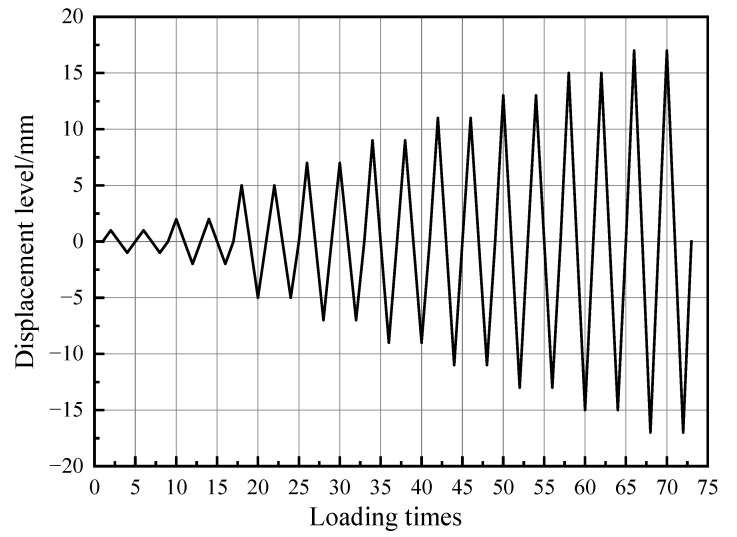
Loading procedures for the specimens.

In consideration of the actual engineering environment, immersed tunnel projects are always subjected to axial pressures. Therefore, axial pressures with two levels, i.e., 300 kN (specimen S1) and 400 kN (specimen S2), were imposed on the specimens. Meanwhile, displacement control was adopted for horizontal cyclic input. According to Figure 8, at each input level, two identical cycles were conducted. At the initial stage, the displacement loading level was 1 mm first, then 2 mm, and finally 5 mm. Whereafter, an increment of 2 mm for each input level was accepted in the tests. According to Li [26], the terminal signal of the tests was set at the loading point that the ultimate capacity reached 85% or severe crushing damage occurred at the shear keys. In order to facilitate the determination of positive and negative loading directions, the push force was defined as positive load, and tension force as classified as negative load.

## 4. Testing Results and Analysis

### 4.1. Failure Mode

**Figure 9:**
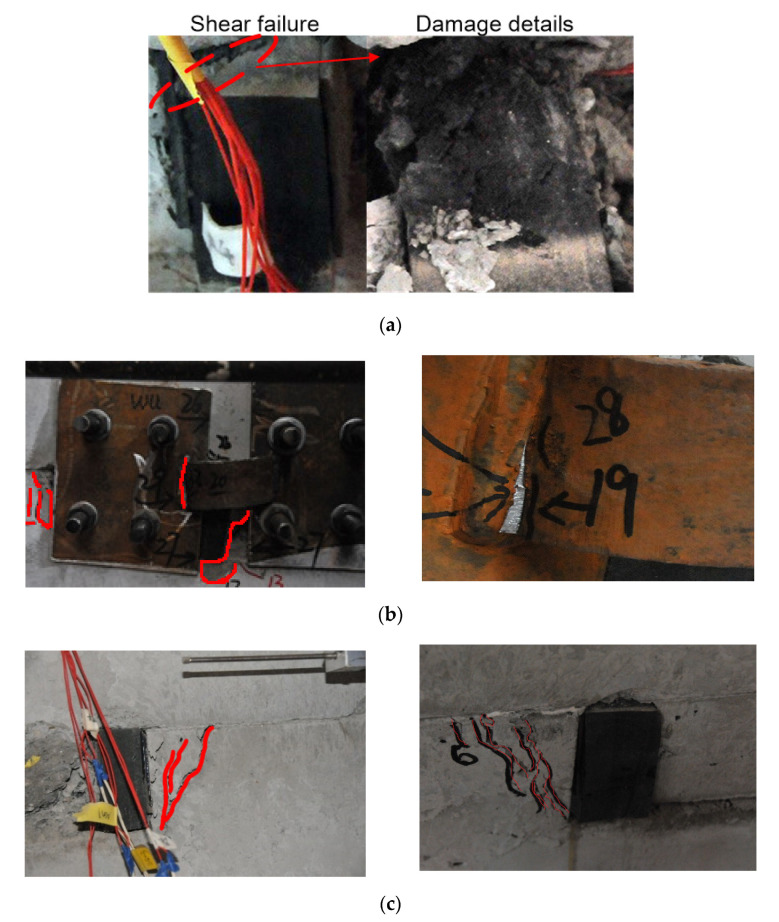
Failure phenomenon: (**a**) Oblique shear failure of the rubber blankets; (**b**) Failure program of the arc-shape energy absorption devices; (**c**) Concrete fracture of the specimens.

**Figure 10:**
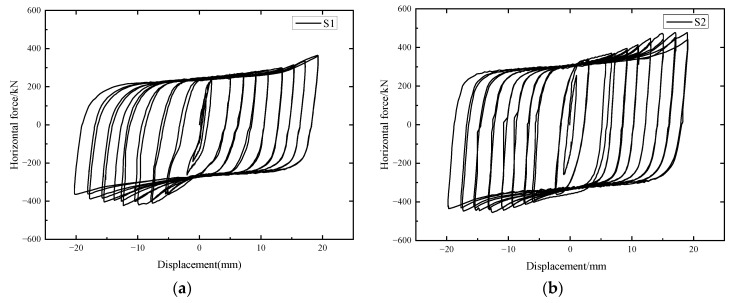
Load-displacement hysteretic curves of the specimens. (**a**) S1; (**b**) S2.

As shown in Figure 9, through the pseudo-static loading tests, the main failure modes of two specimens were observed. The buckling failure of the energy absorption device, shear damage to the shear keys, and concrete crushing occurred in both specimens. The detailed destruction process was as follows:

(1) Similar to the actual immersed tunnel project, the gaps between the shear key and its neighboring major structure portions were filled by the rubber blankets in the tests. Deformation firstly occurred at the rubber blankets with retrospect to cyclic horizontal loading. Then, the deformation of the rubber blankets increased with the increase in load. Finally, oblique shear failure was observed at the central position of the rubber blankets arranged at the gaps between shear keys (Figure 9a);
(2) Since a certain deformation occurred in the rubber blankets, the arc-shaped energy absorption devices got involved quickly (which also can be verified by Figure 10). The arc-shaped energy absorption devices had undergone the local buckling first, and then the failure of the core metal plate emerged (Figure 9b);
(3) After the large-scale deformation occurred in both the rubber blankets and the arc-shaped energy absorption devices, the horizontal shear resistance of the model was mainly provided by the shear key. Concrete cracks with inclination angles in the range of 45–65° with respect to horizontal direction had been marked in the shear keys. Simultaneously, some cracks were also observed on the left and right sides of the bottom “U”-shaped portion (Figure 9c).

### 4.2. Load-Displacement *Hysteretic Curves*

Hysteretic load-displacement curves can not only directly reveal the deformation characteristics with different loading levels of specimens but also reflect the seismic performances of specimens in the areas surrounded by hysteretic loops. Figure 10 presents the hysteretic load-displacement curves of specimens S1 and S2.

The hysteretic load-displacement curves show four working stages: the initial loading stage, the arc-shape energy absorption device buckling plateaus, the concrete cracking stage, and the recession stage. The ultimate load-bearing capacity of specimen S1 was smaller than that of specimen S2, which indicated that the axial pressure can increase the horizontal load-bearing capacity to a certain degree.

As compared with the research results recorded by [30], the loops in the hysteretic curves of the specimens reinforced by the arc-shaped energy absorption devices in the study are plumper than those of a traditional shear key specimen without energy absorption devices reinforcement. Moreover, owing to the protective effects provided by the arc-shape energy absorption devices in this study, the appearance of concrete cracks in the shear keys was effectively delayed.

As the horizontal actuator provided pushing forces to the “T”-shaped segment, horizontal forces were transmitted through shear keys and rubber blankets between them, so the primary deformations were reflected by the rubber blankets. Linear elastic deformations of the rubber blankets were observed firstly, which also can be proved by the narrow hysteretic loops of the specimen under the small displacement loading levels. When subjected to the horizontal displacements of more than 5 mm, the load-carrying effects of the arc-shaped energy absorption device were significant; meanwhile, the hysteretic loops also turned plump. When the local buckling emerged at the arc-shaped energy absorption device, there was a difference in the horizontal stiffness between the two repetitive loops under the same loading grade. The energy absorption effect was distinct. Then, the shear keys participated in the horizontal load-bearing process. The nonlinear behaviors of the specimens were obvious since concrete cracks occurred at the corner position in the shear keys. After more cracks developed, the reaction forces of the specimens first reached peak values and then tended to decrease.

There is general uniformity in the hysteretic performances of the two specimens, in addition to the reaction forces. When compared to hysteretic curves of specimen S2, there has no apparent recession stage of the positive forces, which may be due to the manufacturing errors in the geometric dimension of the testing segments. Moreover, the horizontal forces of specimen S2 were larger than those of specimen S1.

### 4.3. Skeleton Curves

**Figure 11:**
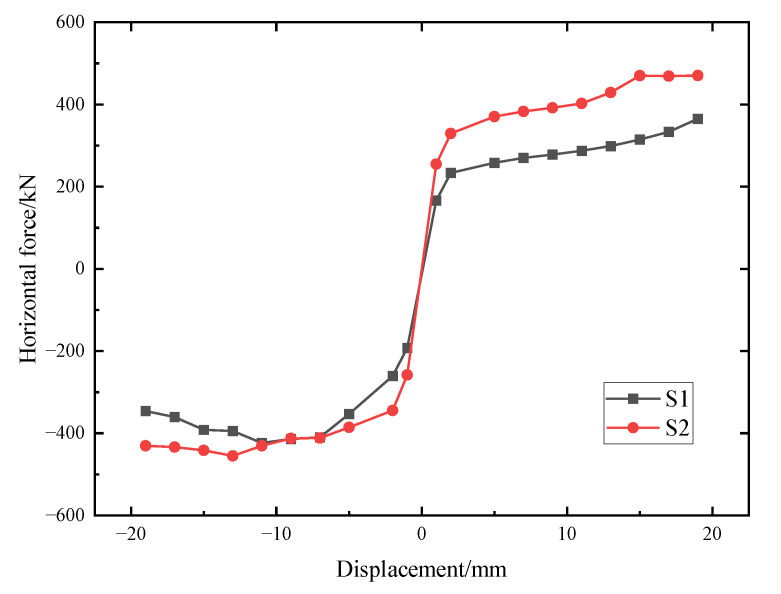
Skeleton curves of the specimens.

Figure 11 presents skeleton curves of the load-displacement hysteretic curves of the specimens S1 and S2. As compared with S1, the load-bearing capacities of S2 were higher. Given that the axial pressure difference between two specimens was 100 kN, specimen S2 bore greater normal stress. As compared with S1, when the same grade of displacement was applied to the specimens, S2 required larger horizontal loads. After specimens reached their ultimate load-bearing capacity, the skeleton curves revealed sudden decreases; this phenomenon was more obvious in the negative direction. The skeleton curves of S1 and S2 also indicated that the load-bearing capacities and ultimate deformation abilities were improved by utilizing the arc-shaped energy absorption devices at the gaps between shear keys compared with the case without the energy absorption devices’ reinforcement. According to the research launched by Park [31], the load-bearing capacity of a specimen can be divided into four types in accordance with loading stages, namely the cracking load, yield load, peak load, and failing load. Based on the skeleton curves in the paper and the testing results recorded by [30], the differences in the load-bearing capacities (cracking load, yield load, peak load, and failing load) between the specimens with and without energy absorption devices’ reinforcement (namely S1 and S0) were 33.0%, 36.7%, 26.0%, and 23.6%, respectively, under the same axial pressure. Furthermore, on the basis of the load-bearing capacities (cracking load, yield load, peak load, and failing load) of S1, the growth ratios between S2 and S1 were 9.0%, 0.4%, 7.4%, and 24.4%, respectively.

### 4.4. Stiffness Degradation

**Figure 12:**
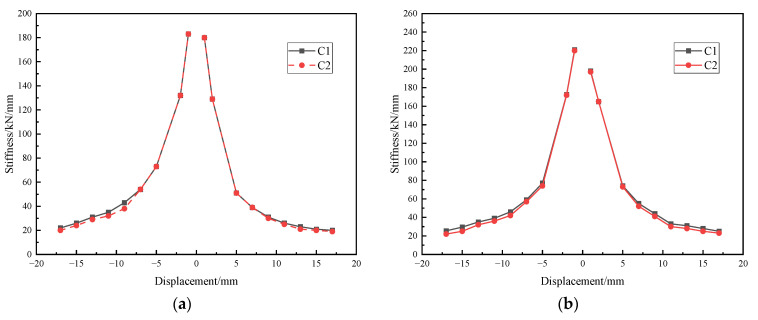
Stiffness degradation curves of the specimens: (**a**) S1; (**b**) S2.

The stiffness was defined as the value of the load-bearing capacity to its corresponding displacement. The stiffness degradation rules are exhibited in Figure 12. Figure 12 indicates that the stiffness degradation curves of the two specimens presented similar forms when subjected to cyclic displacement inputs.

Given that the materials, geometric dimension, and energy absorption devices were the same, an increase of the axial pressure from 300 kN to 400 kN apparently improved the initial stiffness of the shear key. Comparing the initial stiffness of S1 with S2, increased ratios in the negative direction and positive directions were approximately 20.0% and 10.0%, respectively. As buckling phenomena occurred at the arc-shaped energy absorption devices, the stiffness in the second cycle, namely C2 in Figure 12, was smaller than that in the first cycle, namely C1, under the same displacement loading. Since concrete cracks turned out, the stiffness difference between the two cycles in the stiffness degradation curves was more distinct. In retrospect [30], there was only a little difference between the initial stiffness in the case with and without the arc-shaped energy absorption devices reinforcement, but an increase in structural stiffness was found at nonlinear stages. Therefore, it can be seen that the axial pressure significantly influenced the stiffness of the shear keys in immersion joints, and the arc-shaped energy absorption devices can improve the stiffness of the shear keys to a certain degree at nonlinear stages. Through the phenomena aforementioned, the specimens S1 and S2 had fully implemented the role of the arc-shaped energy absorption devices. Both reinforced testing models exhibited good deformation adaptability which is a crucial requirement of an immersed tunnel engineering project.

It should be noted that the flexible adjustments are an important feature of an immersion joint; thus, accessional energy absorption devices must not limit the allowable displacement of immersion joints when subjected to earthquake events. During the anti-seismic design process of immersion joints, a reasonable adjustment for the stiffness of an energy absorption device is required.

### 4.5. Shock Absorption Ability

The seismic energy dissipation ability can be calculated by enclosed areas under the load-displacement hysteretic curves. According to the enclosed areas from Figure 10, the accumulated energy dissipation values of S1 and S2 were 236,032 kN.mm and 241,601 kN.mm respectively, which denoted that the axial pressure could affect the energy dissipation of the specimens.

**Figure 13:**
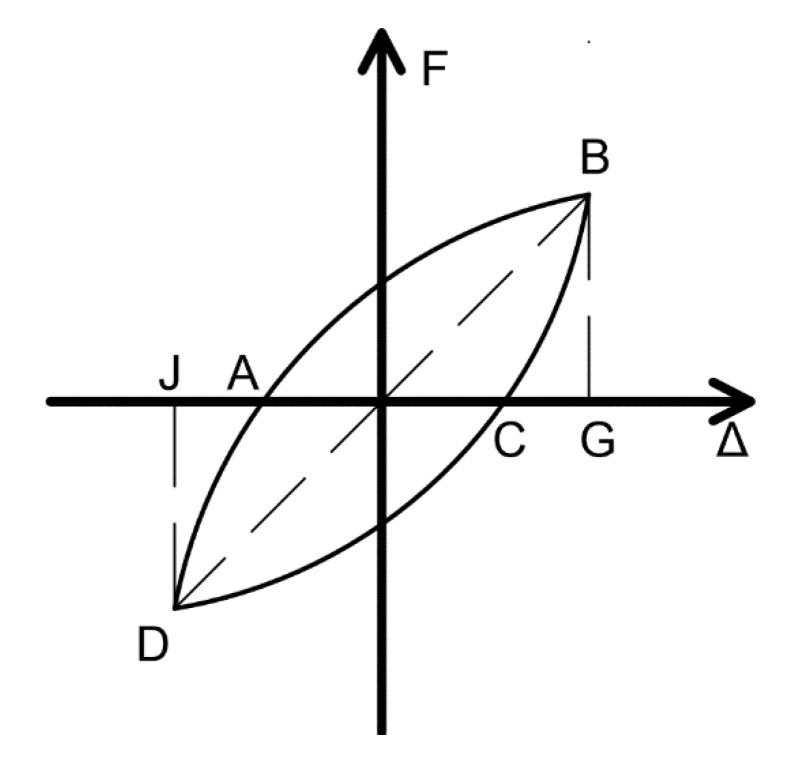
Definition of β.

According to the results of single loops from the hysteretic curves under different displacement inputs, the equivalent viscous coefficient value β [29] can be measured as shown in Figure 13, where ∆ denotes the displacement of the specimen and F means the load-bearing capacity of the specimen), which can be defined as:

(1) $$\beta \;=\;\frac{1}{2\pi }\frac{S_{ABCD}}{S_{\Delta OBG}+S_{\Delta ODJ}}$$

where $S_{ABCD}$ means the aera of ABCD, $S_{\Delta OBG}$ means the aera of triangle OBG, $S_{\Delta ODJ}$ means the aera of triangle ODJ.

According to hysteretic curves of S0 [30], S1, and S2, the equivalent viscous coefficient values corresponding to the cracking stage, yield stage, deformation development (peak load) stage, and failing stage of all the specimens were calculated and listed in Table 3. The equivalent viscous coefficient value decreased with the development of structural deformation, which implies that the increase in displacement input can result in accumulated damage to the testing specimens. Comparing the equivalent viscous coefficient values of S0 [30], S1, and S2, the equivalent viscous coefficient values of S2 were roughly equivalent to those of S1, while the equivalent viscous coefficient values observed from S0 were smaller than those of S1 and S2. The phenomena indicate that the arc-shaped energy dissipation devices lead to an increase in the equivalent viscous coefficient of the specimens.

### 4.6. Ductility

**Figure 14:**
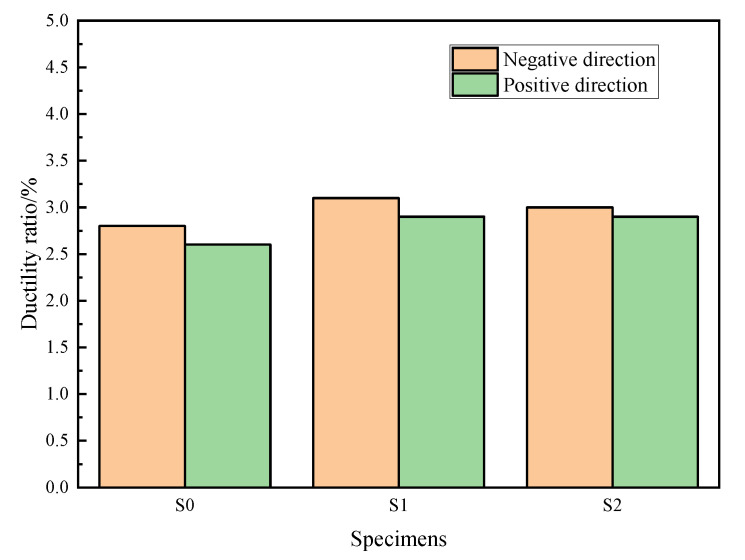
Ductility ratio values of the specimens.

The ductility ratio of displacement can reflect the anti-seismic capacity of a structure; the ductility ratio in the study was defined as the ratio of the ultimate displacements of the specimen to length of the shear key. As there was a certain displacement difference between the negative and positive directions, Figure 14 presents the ductility ratio values of the case with and without arc-shaped energy absorption devices [30] in both directions. Through Figure 14, it can be seen that the arc-shaped energy absorption device had a positive influence on the ductility ratio of the shear key. Furthermore, owing to an increase in the axial pressure of S2, the ductility ratio in the negative direction of S2 was smaller than that of S1, which denotes that the axial pressure had a negative influence on the ductility ratio of the shear key.

### 4.7. Discussion

According to the Chinese specifications for seismic design of highway bridges [32], special attention should be given to the ductility seismic design of underground structures. When the toughness of an immersion joint material is insufficient to absorb the shear deformation caused by the stratum on the cross section, necessary anti-seismic or energy absorption measures must be considered during the design process. Meanwhile, it is very difficult to accurately provide the immersion joint displacement control index suitable for different immersed tunnel projects, so the joint deformation of immersed tunnels in strong earthquake zones should be limited. Seismic mitigation and the energy-dissipation method can meet the requirements of disaster prevention and mitigation of immersed tunnels. In traditional design, the load-bearing capacity of an immersion joint is assumed to be the load-bearing capacity of the shear keys. However, the aforementioned analysis indicates that the ultimate load-bearing capacity of the model is contributed by the shear key, rubber blankets, and arc-shaped energy absorption devices. Therefore, the appropriate stiffness and seismic mitigation ability of energy absorption devices should be taken into consideration during the anti-seismic design of immersed tunnels. Excessive stiffness and immoderate flexibility of an energy absorption device should be prohibited.

## 5. Conclusions

To discover and verify the feasibility of a novel energy absorption device for shear keys in an immersion joint and investigate the effect of axial pressure on the seismic behavior of the shear keys which were strengthened by the arc-shaped energy absorption devices, a series of pseudo-static tests were carried out. Based on a comprehensive analysis of testing results, the following conclusions can be drawn:

(1) Under the horizontal cyclic displacement loading, the horizontal forces were mainly from a synergistic effect of the rubber blankets, arc-shaped energy absorption devices, and shear keys. The failure mode of the testing specimens indicated that the arc-shaped energy absorption devices failed earlier than the shear keys. As a consequence, the arc-shaped energy absorption devices applied to the shear keys can prevent the shear keys from premature failure.
(2) The shear key enhanced by the arc-shaped energy absorption devices exhibited better anti-seismic performances than an ordinary shear key. Loops in the hysteretic curves of S1 and S2 were plumper than those from S0. Comparing the load-bearing capacity of the shear key model with and without energy absorption devices reinforcement, the differences in the cracking load, yield load, peak load, and failing load were 33.0%, 36.7%, 26.0%, and 23.6%, respectively. The equivalent viscous coefficient values observed from S0 were smaller than those of S1 and S2.
(3) The testing results revealed that as the axial pressure increased from 300 kN to 400 kN, obvious differences were observed in the load-bearing capacity, accumulated energy dissipation value, and initial stiffness. The growth ratios of load-bearing capacities (cracking load, yield load, peak load, and failing load) between S1 and S2 were 9.0%, 0.4%, 7.4%, and 24.4%, respectively. The accumulated energy dissipation values of S1 and S2 were 236,032 kN.mm and 241,601 kN.mm respectively. As for the initial stiffness, a comparison between S1 and S2 exhibited an increased ratio in the negative direction of approximately 20.0%. While the axial pressure had little influence on the equivalent viscous coefficient value and ductility ratio of the reinforced shear keys (through a comparison of the equivalent viscous coefficient value of S1 and S2, it was found that the maximum error was 0.01).
(4) Analytical data also proposed that the deformation capacity of the energy absorption device should meet the limit requirement of relative displacement of the joint recommended by the related standards, and the combined shear stiffness of the rubber blankets, shear keys, and energy absorption devices also should be noted during the anti-seismic design process of an immersion joint.
(5) The arc-shaped energy absorption device can provide theoretical basis and scientific support for further development of a new type of anti-seismic immersion joint and facilitate the application of tunnel shock control technology in engineering practice. However, this study was primarily focused on the energy absorption device made from Q235 metal, so further studies on the parameter optimization of the arc-shaped energy absorption device and other energy-absorptive material used for anti-seismic design of immersion joints are required.

## Figures and Tables

**Table 1:** Mechanical parameters of steel plate.

Thickness/mm	Type	Yield Strength/MPa	Extension Stength/MPa	Elastic Modulus/GPa	Yielding to Tensile Strength Ratio
10	Q235	310	428	200	0.73

**Table 2:** Specific parameters of specimens.

Specimens	Concrete Mark	Steel Grade	Axial Pressure
S1	C50	HRB400	300 kN
S2	C50	HRB400	400 kN

**Table 3:** Equivalent viscous coefficient values.

Tests	β			
	Cracking Stage	Yield Stage	Deformation Development Stage	Failing Stage
S0	0.32	0.31	0.28	0.26
S1	0.37	0.36	0.32	0.28
S2	0.38	0.36	0.33	0.29

## Data Availability

Not applicable.
